# Laboratory Experiments and Numerical Simulation Study of Composite-Material-Modified Loess Improving High-Speed Railway Subgrade

**DOI:** 10.3390/polym14153215

**Published:** 2022-08-08

**Authors:** Li Luo, Xingang Wang, Chen Xue, Daozheng Wang, Baoqin Lian

**Affiliations:** State Key Laboratory of Continental Dynamics, Department of Geology, Northwest University, Xi’an 710069, China

**Keywords:** loess, composite material, improvement, high-speed rail, subgrade settlement

## Abstract

Construction of high-speed railway subgrade on loess soils in the Loess Plateau is risky because such soil is susceptible to differential settlements. Various soil-improvement methods have been used to enhance the mechanical properties of loess. Lime-ash soil and cement-lime soil are the most commonly used methods in the improvement of loess subgrade, while few studies have been found on loess subgrade improvement by using composite material consisting of traditional materials and new materials. A series of direct shear tests and unconfined compressive tests were conducted on the loess specimen with the addition of three kinds of composite materials: traditional material cement, new material polypropylene fiber and SCA-2 soil curing agent. The numerical simulation was conducted on loess subgrade in an actual engineering practice. The experimental results show that cement, polypropylene fiber and SCA-2 soil curing agent can effectively improve the shear strength and compressive strength of loess, and the influence degree is cement > fiber > curing agent. Additionally, based on the relative strength characteristics of the improved loess, an optimal improvement scheme for the composite-material-modified loess was obtained: 16% cement content + 0.5% fiber content + 4% curing agent content. The numerical simulation results revealed that the compressive strength index of the improved loess has a significant impact on the subgrade settlement, and the optimal improvement scheme obtained from comprehensive analysis can effectively improve the settlement of high-speed railway subgrade under vibration load.

## 1. Introduction

**Figure 1:**
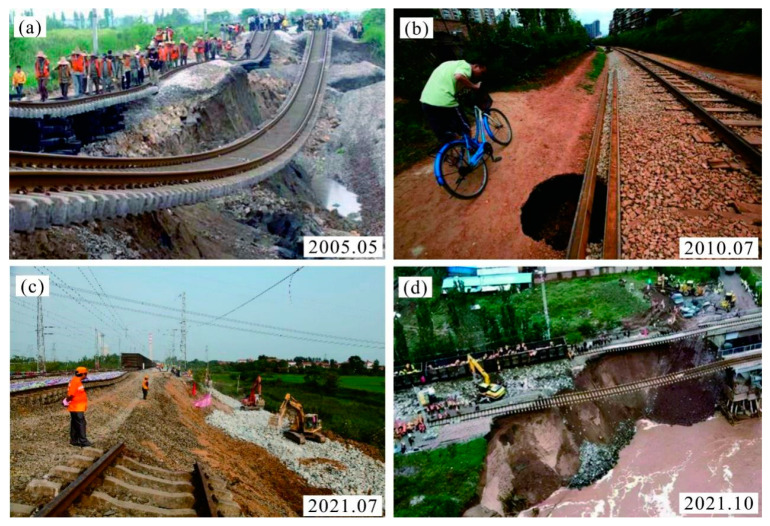
Typical high-speed railway foundation settlement disasters in China: (**a**) Xiaoyong railway collapse disaster; (**b**) Kunming railway collapse disaster; (**c**) Longhai railway collapse disaster; (**d**) South Tongpu railway collapse disaster.

High-speed railway subgrade has been identified as one type of an important foundation of bearing track structures and trains [1,2,3]. It is known that excessive settlement of the subgrade will cause serious disasters such as subgrade collapse and train derailment (Figure 1); however, the millimeter-level settlement-control standard of high-speed railway lines poses unprecedented challenges to subgrade engineering, which is a special issue for loess soils in loess areas of China [4].

Loess soils are characterized by a metastable structure, high porosity, water sensitivity and being loosely compacted [5,6,7], which leads to a high risk of disaster under external forces [8,9,10]. Thus, constructions of high-speed railway in loess areas are prone to uneven settlement of high fill embankment, subgrade slope instability and other engineering disasters [11,12]. Therefore, effectively improving the loess and controlling the settlement and deformation of the subgrade has become a significant problem that needs to be solved urgently in the current high-speed railway construction. At present, it is recognized that the improved subgrade has been widely used in high-speed railway engineering [13,14]. Japan, Germany and other countries have clearly stipulated that the railway subgrade could be improved by cement, lime and other materials. For the improvements of soil subgrade, the common treatment methods include physical and chemical methods. Generally, the physical improvement method is a way to change soil gradation by adding coarse particles such as sand and gravel [15], and the chemical improvement is usually employed by mixing cement [16], lime [17,18] and polypropylene fiber [19,20] into the soil.

In recent years, research scholars have carried out a series of studies on the improvement of subgrade in loess areas by using traditional materials including cement, lime, etc. and by using new improvement materials such as polypropylene fiber, curing agent, etc. By conducting field compaction tests on loess with different lime contents, Zhang et al. [21] investigated the effects of moisture content, lime content and compaction strength on the compaction characteristics of loess subgrade. The results show that the improvement effect of lime on loess increases with water content. Jiang et al. [22] studied the influence of cement content, compaction coefficient and curing time on the mechanical strength index of cement-modified loess (CML) compacted by vertical vibration compaction (VVCM), and finally established a model to predict the growth law of strength characteristics of CML. With the general improvement of environmental protection awareness, new environmentally friendly improvement materials have been used in soil improvement [23,24,25]. By extracting lignin from waste paper to improve loess with different contents, and carrying out triaxial tests and SEM observations on the modified loess, Wang et al. [26] found that when the lignin content was 4%, the modified loess had the best liquefaction resistance and compactness. Wang et al. [20] studied the role of polypropylene fiber played as a reinforcing material to enhance the mechanical properties of loess. Through ring shear tests, the optimum content of polypropylene fiber to improve loess properties was found to be 0.5%.

Previous studies revealed that the mechanical properties such as compressive strength [27,28] and shear strength [29] of the improved soil are significantly enhanced, which thus can effectively decrease the settlement deformation of high-speed railway subgrade under external load. Additionally, it has been widely recognized that the improvement of soil mass by adding a variety of composite materials with a certain mixing ratio is better than that by adding a single material [30,31]. At present, the addition of lime-ash or cement-lime mixes to soil is the most-used method for the improvement of loess subgrade, while few studies were found on the improved materials improve loess subgrade by combining new materials with traditional materials [32].

In this study, to explore a new method of loess subgrade improvement, traditional improvement materials (cement) and new improvement materials (polypropylene fiber, SCA-2 soil curing agent) were selected to form composite materials with different contents to improve loess. By conducting a series of laboratory test and orthogonal analysis, the influence of composite material dosage on the strength index of improved loess was studied. Additionally, the influence of different strength indexes of the improved loess on subgrade settlement was also discussed by conducting numerical simulation. Finally, the best scheme of the composite material to improve loess was obtained, which can provide new ideas and experimental reference for the improvement of high-speed railway subgrade in loess areas.

## 2. Materials and Methods

### 2.1. Materials

**Figure 2:**
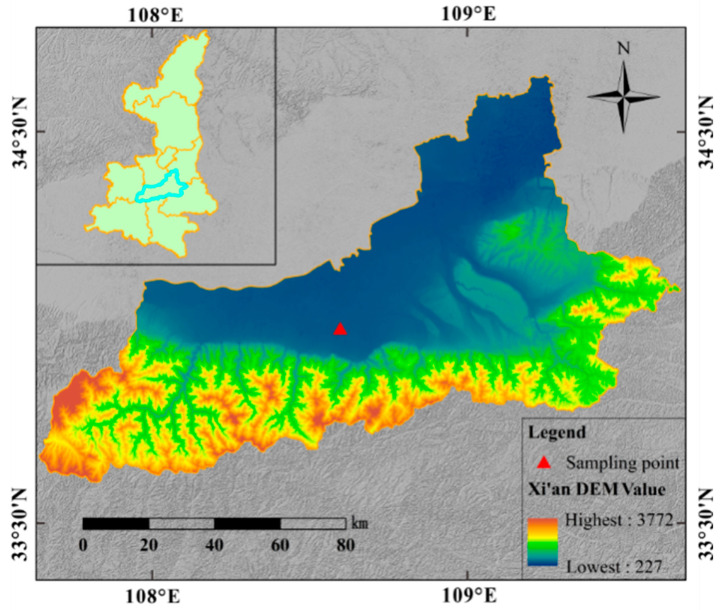
Location map of the sampling site.

**Figure 3:**
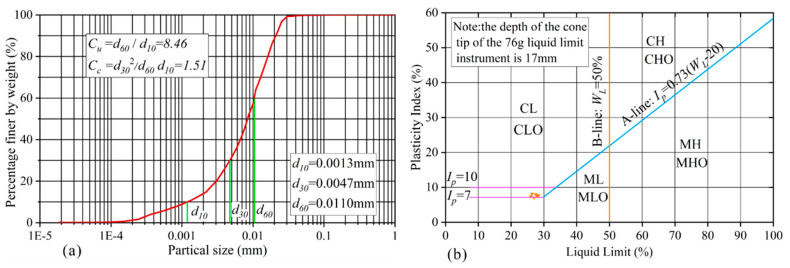
Physical properties of loess specimens showing: (**a**) grain-size distribution curve; (**b**) Casagrande plasticity chart.

The loess samples were collected from a high-speed railway subgrade in Xi’an, Shaanxi Province of China (Figure 2). The grain-size distribution curve of soil sample was determined by conducting sieve analysis (Figure 3a). The basic physical indexes including dry density, water content, specific gravity, liquid limit (WL) and plastic limit (Wp) shown in Table 1, were determined according to the Chinese National Standards (CNS) GB/T50123–1999 [33]. The results reveal that the soil sample has a relatively low water content and low dry density. According to the Casagrande plasticity chart shown in Figure 3b, the loess is classified as a low-plastic clay. Three improvement materials used in the test are as follows: the ordinary portland cement with a strength grade of 42.5 [17], the chemical composition of which is shown in Table 2; the bundle monofilament polypropylene fiber with 6 mm length [31], the main physical and mechanical properties of which are shown in Table 3; and the SCA-2 soil curing agent (dark gray) is produced in Qingdao, China, which is a polymer organic liquid curing agent with pH value less than 1, strong cohesiveness, high-concentration liquid and density ranging from 1.30 to 1.35 g/cm^3^.

### 2.2. Experimental Program and Procedure

In this study, the effects of the composite material composition on the shear strength and compressive strength of loess soil were investigated by conducting direct shear and unconfined compression tests on soil samples added with different amounts of improving materials, i.e., cement, polypropylene fibres and curing agent SCA-2. The contents of added materials were expressed on a dry basis of loess soil and varied from 0 to 16% for cement, from 0 to 1% for fibres and from 0 to 16% for the curing agent [24]. The content of materials is controlled by the ratio of the materials amount to the dry loess amount. The orthogonal test method was used to design the test scheme, which can discuss the influence of each test factor on the strength index under the premise of lower test quantity [34]. L_25_ (5^6^) orthogonal table was adopted in this test, and there were 25 combination ratio schemes (Table 4).

**Figure 4:**
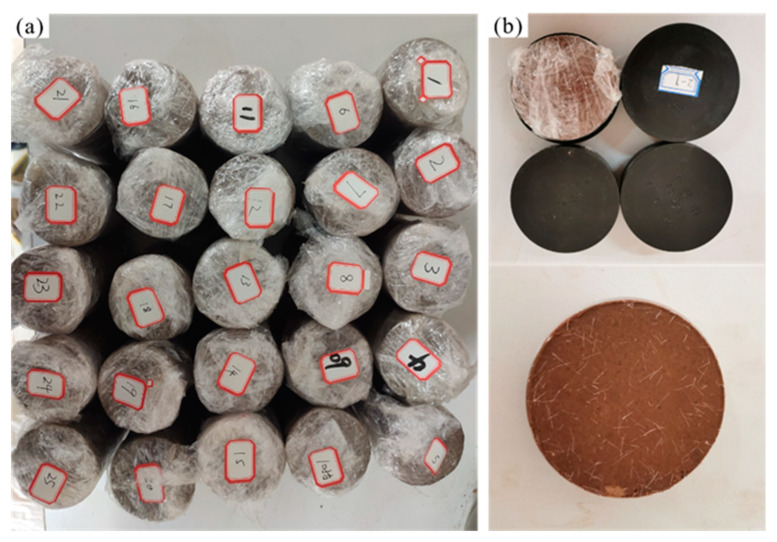
The improved loess samples: (**a**) cylindrical samples; (**b**) ring knife samples.

During sample preparation, the cement, fiber and curing agent were mixed according to the established ratios listed in Table 4. To be more specific, the mixture was added in sequence (first cement, then fiber, then curing agent). After that, water was added into the mixed soil samples according to the optimal moisture content of loess (14%) by compaction test. Finally, different samples were prepared by sample preparation instrument. Each group sample includes two kinds of samples: four ring-knife samples with a diameter of 61.8 mm and height of 20 mm were prepared, and a cylindrical sample (i.e., approximately 50 mm in diameter and 100 mm in height) was prepared (Figure 4). The compaction coefficient K of the prepared samples was controlled to be 0.93. The prepared samples were wrapped in plastic and stored in a sealed container that prevents evaporation for about 7 days before conducting tests to obtain uniform water content within samples [22].

## 3. Results

To study the influence of the various test factors on the strength indexes of loess, the extreme difference analysis and the variance analysis methods were applied to the results of direct shear stress and unconfined compressive tests. In addition, the influences of various factors on the internal friction angle, cohesion and compressive strength of loess were also discussed.

### 3.1. Test Results

**Figure 5:**
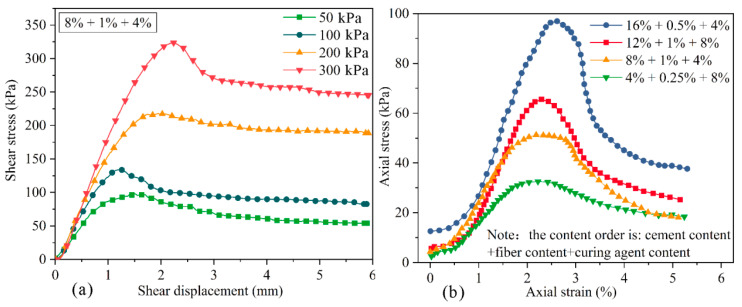
Test results of some samples: (**a**) shear stress–shear displacement curve of direct shear tests; (**b**) axial stress–strain curve of unconfined compressive tests.

Due to the limited space, the test results of some samples were selected in Figure 5. The peak shear stress of improved loess in the direct shear tests was identified as the failure shear strength when the peak shear stress emerged. Conversely, without the emergence of the peak shear stress, the shear stress corresponding to 15% strain was determined to be the shear strength. Then, the internal friction angle and cohesion of modified loess can be obtained by Mohr–Coulomb theory. The axial stress–strain curve of the unconfined compressive strength test of some samples is shown in the Figure 5b; the stress–strain curve showed a strain-softening trend, so the maximum axial pressure was identified as the unconfined compressive strength of the improved loess. Additionally, if the stress–strain curve showed a strain-hardening trend, the stress was taken as the unconfined compressive strength of the sample when the axial strain was 15%.

### 3.2. The Extreme Difference Analysis Results

The extreme difference analysis is an important method in the analysis of orthogonal test results [35], which can represent the average value of the test index (i.e., friction angle, cohesion and compressive strength) corresponding to a single factor at a specific level. The change under the same conditions reflects the different levels of influence impacting the test indexes. By comparing the extreme difference average value R (difference between maximum and minimum average values under different contents) of test indexes, the influence degree of each factor on the test index was directly obtained. It is worth noting that the greater R value demonstrates the greater influence of this factor on the test index.

**Figure 6:**
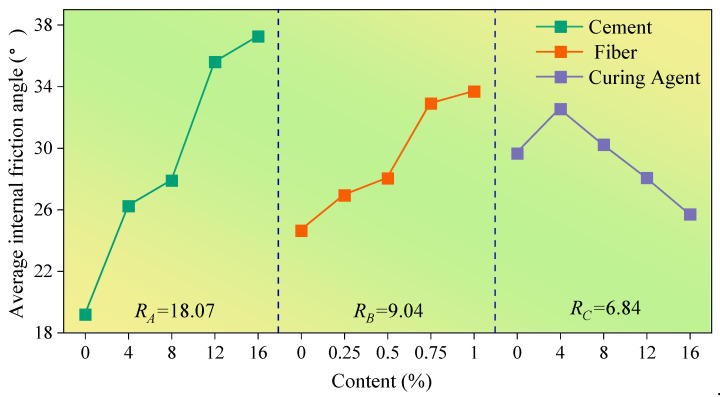
The influence of various factors on the internal friction angle.

**Figure 7:**
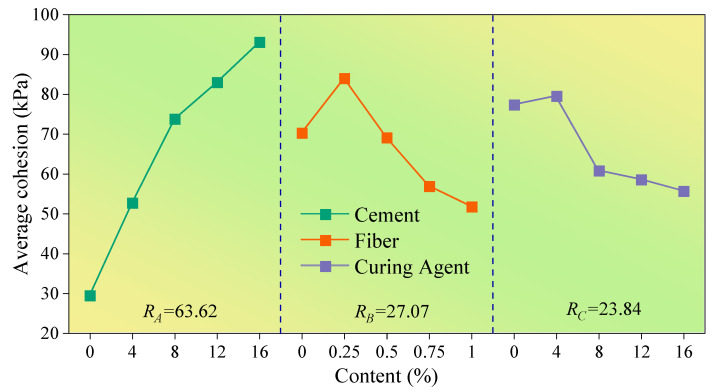
The influence of various factors on the cohesion.

**Figure 8:**
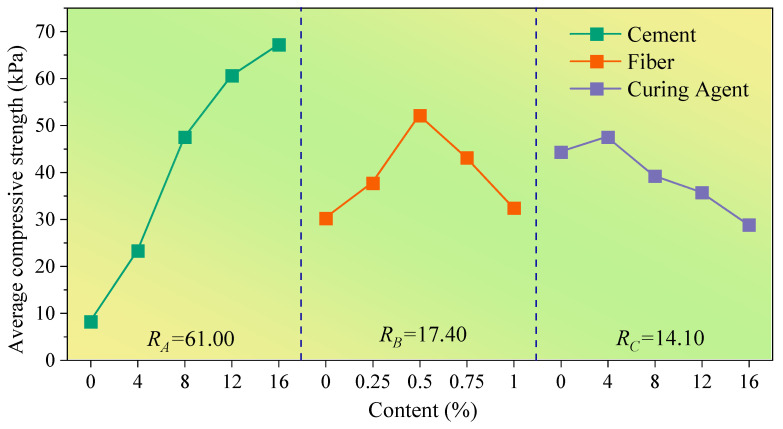
The influence of various factors on the compressive strength. **Notes**: *R_A_, R_B_* and *R_C_* represent the extreme difference of each strength index under different contents of cement, fiber and curing agent, respectively.

To more intuitively analyze the influence level of various factors on test indexes, the content of cement, fiber and curing agent was taken as the abscissa, and the average value of the test index corresponding to the addition of cement, fiber and curing agent with different content was set as the ordinate; the influence trend diagram of the test result index under different levels of each factor is shown in Figure 6, Figure 7 and Figure 8.

The influence of various factors on the relevant mechanical properties of the improved loess is shown in Figure 6, Figure 7 and Figure 8. By comparing the average value R, the level of influence of cement, polypropylene fiber and curing agent on the internal friction angle, cohesion and compressive strength was obtained as follows: cement > polypropylene fiber > SCA-2 soil curing agent. Additionally, it can be found that the cement content is positively correlated with the average value of the internal friction angle, cohesion and compressive strength of improved loess, and with the increase in curing-agent content, the average strength index of improved loess increases at first and then decreases, reaching the maximum value when the curing agent content is about 4%. However, the variations of internal friction angle, cohesion and compressive strength of loess soils with polypropylene fibers are quite different; the average value of internal friction angle increases continuously with the increase in fiber content, while the average cohesion and compressive strength increase first and then decrease with the increase in fiber content, and reach the maximum when the fiber content is approximately 0.25% and 0.5%, respectively.

### 3.3. The Variance Analysis Results

It must be considered that range analysis can directly analyze the impact of test factors on the results, but ignores the impact of error [36]. To better analyze test results, data-analysis software SPSS was employed to conduct variance analysis on the results. In this software, *p* (when *p* < 0.05, it means that the factor has a significant influence on the dependent variable) was analyzed. Therefore, the significance of the influence factors on the test index was investigated, which can provide reference for the study of the optimal content of each experimental factor.

The variance analysis results (Table 5, Table 6 and Table 7) show that the order of influence of cement, polypropylene fiber and curing agent on internal friction angle, cohesion and compressive strength is as follows: cement > fiber > curing agent, which is consistent with the results obtained from the extreme difference analysis (Figure 6, Figure 7 and Figure 8). By comparing the significance *p* value, it can be found that cement has a great influence on the internal friction angle, cohesion and compressive strength of improved loess, while polypropylene fiber has a greater influence on the internal friction angle and compressive strength compared with cohesion. Additionally, little effect of SCA-2 curing agent on the internal friction angle, cohesion and compressive strength of improved loess was found.

According to the extreme difference analysis results and the variance analysis results, it is found that the maximum value of internal friction angle, cohesion and compressive strength of the improved loess was achieved by the addition of 16% cement, 4% SCA-2 soil curing agent and 1–0.25% polypropylene fiber. 

## 4. Numerical Simulation of Settlement of High Railway Foundation

In this paper, MIDAS/GTS finite-element-analysis software was used to simulate the settlement of high railway foundation in actual engineering practice. The improvement scheme obtained from the test results was used to improve the subgrade, and the settlement of the improved subgrade under the vibration load of high-speed railway was compared, then the best improvement scheme for improving the railway foundation in the loess area was obtained and the feasibility of the loess improvement scheme was also tested.

### 4.1. Model Description

**Figure 9:**
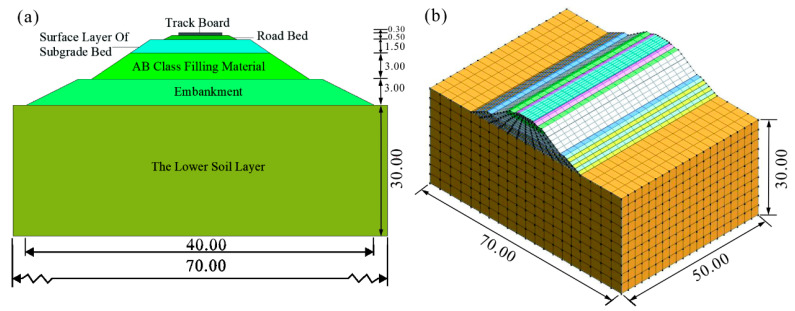
Model diagram of high-speed railway subgrade. (**a**) Schematic diagram of the standard section of subgrade (unit: m); (**b**) 3D model of high-speed railway subgrade (unit: m).

According to the Chinese national technical standard “Standard of Railway Subgrade Design” (TB10001–2016) [37], the finite element model of train dynamic subgrade is established; the section length and height of the model are 70 m and 38.3 m, respectively (Figure 9a), and the extension length of subgrade is 50 m (Figure 9b). The numerical simulation model is divided into 6920 units and the boundary is viscoelastic. The embankment thickness of the high-speed railway subgrade is 3 m, which is the main part of subgrade improvement. The way to improve the embankment was to use the construction method of layered filling; the embankment was divided into six layers, with each layer being 50 cm high, and the improved loess in the embankment was sandwiched by loess. 

**Figure 10:**
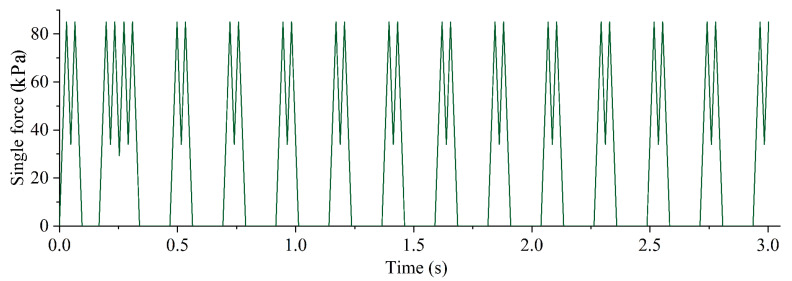
Time-history curve of excitation force load.

To simulate the vibration load caused by the train running, the moving axle load of periodic loading and unloading was applied to the high railway foundation model (Figure 10). The calculated high-speed rail speed is 300 km/h. The train time history analysis module in Midas numerical simulation software was used, the simulation results data were assessed over an interval of 0.03 s and the variation in the settlement in the duration of 3 s was analyzed when the train passed by.

The high-speed railway subgrade is mainly divided into the following parts: track slab, road bed, surface layer of subgrade bad (graded gravel), bottom layer of subgrade bad (A B fillers), embankment (improved part) and lower soil (loess). According to the results of the orthogonal test, three improvement schemes were obtained, which gave priority to the influence of the internal friction angle (16% + 1% + 4%), cohesion (16% + 0.25% + 4%) and compressive strength (16% + 0.5% + 4%), respectively, and these schemes were denoted by schemes X, Y and Z, respectively (the content order of the improved scheme is: cement content + fiber content + curing agent content). To acquire the optimal fiber content, the modified scheme W (16% + 0.75% + 4%) was added. The relevant numerical simulation parameters of the above materials were obtained through laboratory tests and literature review [38]. The cohesion and internal friction angle of loess and improved loess were obtained by using the Coulomb–Mohr theory through direct shear tests, and the elastic modulus was obtained from the results of unconfined compressive strength tests. The calculation parameters of the finite element model are shown in Table 8.

### 4.2. Numerical Simulation Results

**Figure 11:**
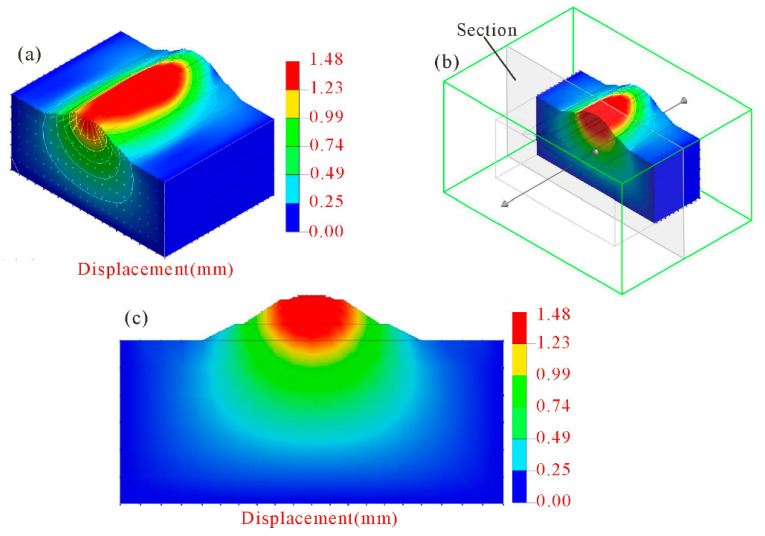
Settlement contour map of unimproved high-speed railway subgrade. (**a**) Three-dimensional contour map; (**b**) schematic diagram of section position; (**c**) section diagram of maximum settlement.

Settlement variation of unimproved high railway subgrade is shown in Figure 11. Using the model section interception method (Figure 11b), the settlement contour map of the section with the greatest subgrade settlement was obtained (Figure 11c). As seen in Figure 11, the settlement phenomenon of the high-speed railway subgrade without improvement is obvious when a single high-speed railway train passes by, with the maximum settlement value of the subgrade reaching about 1.48 mm.

**Figure 12:**
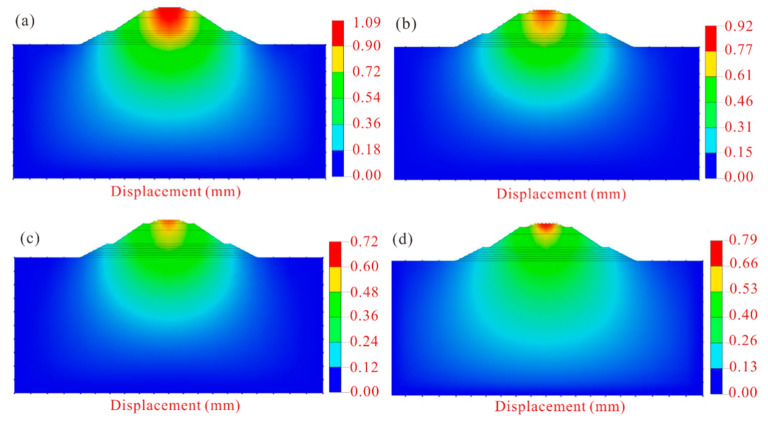
Settlement contour map of high-speed railway subgrade after improvement. (**a**) Scheme X; (**b**) scheme Y; (**c**) scheme Z; (**d**) scheme W.

The changes in settlement of high-speed railway subgrade after improvement are shown in Figure 12. After the improvement of the scheme X, the settlement of high-speed railway subgrade is greatly decreased, and the maximum settlement drops to 1.09 mm, which is 0.37 mm lower than that of the unimproved high-speed railway subgrade. The maximum settlement of the high-speed railway subgrade improved by the scheme Y is 0.92 mm, which is 0.54 mm lower than that of the unimproved subgrade. The improvement effect of scheme Z is pronounced, with the maximum settlement only 0.72 mm, which is 0.74 mm less than that of the unimproved high-speed railway subgrade. The maximum settlement of the high-speed railway subgrade improved by the scheme W is 0.79 mm, which is 0.67 mm less than the unimproved subgrade. Clearly, the improvement scheme Z has the best effect on the improvement of high-speed railway subgrade, thus the best improvement scheme for improving the high-speed railway foundation with composite materials is obtained: 16% cement content + 0.5% fiber content + 4% curing agent content.

## 5. Discussion

### 5.1. Influence Mechanism of Different Materials on the Strength Properties of Modified Loess

**Figure 13:**
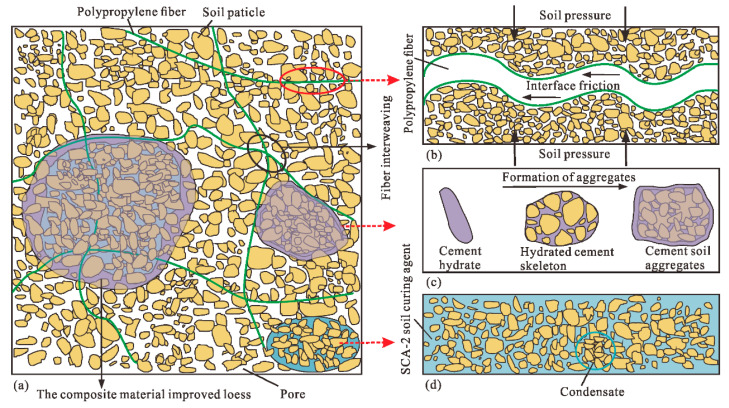
Microscopic schematic diagram of the mechanism of composite materials improving loess (modified from Yan et al. 2021). (**a**) General schematic diagram; (**b**) polypropylene fiber; (**c**) cement; (**d**) SCA-2 soil curing agent.

Through direct shear test and unconfined compressive strength test, it can be found that the improvement effect of cement, polypropylene fiber and SCA-2 soil curing agent on the internal friction angle, cohesion and compressive strength of modified loess is quite different. The reason is that the three modifying materials have different ways of interacting with loess [39], and the influence degree on the relevant strength characteristics of loess is also quite different (Figure 13).

The addition of cement will react with water to produce cement hydrate [40] that forms a cement-soil skeleton between soil particles [29], which makes the soil particles more closely connected and increases the friction resistance on the surface of the particles. With the increase in cement content, cement hydrate even generates gel to wrap soil particles to form aggregates (Figure 13a,c), which makes the soil cemented and hardened. Therefore, this can further explain the phenomenon in Figure 6, Figure 7 and Figure 8 that the internal friction angle, cohesion and compressive strength of the modified loess increase continually with the increase in cement content.

As shown in Figure 13a,b, the random distribution of polypropylene fibers in soil can form a three-dimensional reinforcement network that is similar to the antislide pile, which can increase the interface friction of soil and restrict the relative movement of soil particles [39,41]. Therefore, the internal friction angle of the modified loess can be greatly increased, and this can further explain the phenomenon shown in Figure 6 that the fiber content and the internal friction angle is positively correlated. However, for the cohesion of modified loess, the addition of a small amount of polypropylene fiber causes friction and interlocking between fibers and between fibers and soil particles [42]. An increase in the fiber content will cause obvious cracks and pores in the soil [20], resulting in a negative effect. Consequently, as seen in Figure 7, when the fiber content is 0.25%, the improved loess has the greatest cohesion. For the compressive strength of improved loess, cement enhances the compressive capacity of loess, and thus the improved loess is prone to brittle fracture. The addition of an appropriate amount of fiber enhances the ductility of the soil [31], and this can further explain the phenomenon illustrated in Figure 8 that the compressive strength of the modified loess is the maximum when the fiber content is 0.5%.

SCA-2 soil curing agent is mainly composed of sodium silicate and polyacrylamide, can chemically react with cement to produce gel, which can wrap soil particles, reduce pores in soil, and thus increase the cohesion and compressive strength of modified loess [43]. However, when the content of curing agent exceeds 4%, the soil produces a saturation reaction to it (Figure 6, Figure 7 and Figure 8), and the excess liquid curing agent fills the pores of the soil and acts as a lubricant, which reduces the strength index of the soil (Figure 13a,d), and this can further explain the phenomenon shown in Figure 6, Figure 7 and Figure 8 that the internal friction angle, cohesion and compressive strength of the modified loess increased firstly with the curing agent content and then decreased from the maximum strength at the content of 4%.

### 5.2. Influence of Loess strength Characteristics on Subgrade Settlement

**Figure 14:**
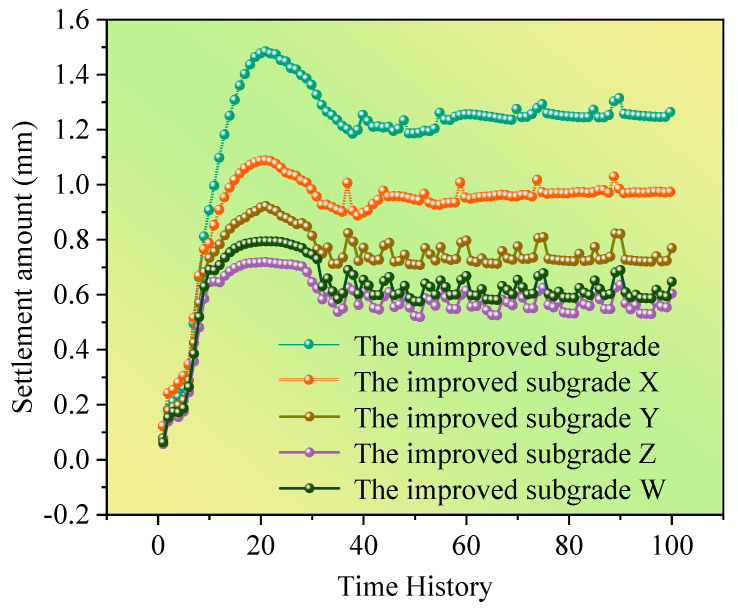
Time-history curve of subgrade settlement.

The influence of the strength characteristics of modified loess on subgrade settlement is complicated. From the experimental results and numerical simulation results, it can be found that the improvement scheme can effectively reduce the settlement of high-speed railway subgrade. Through analyzing the settlement change curve of subgrade after the train passes by (Figure 14), three stages of subgrade settlement were identified: the instantaneous settlement stage, in which the settlement of high railway subgrade under train dynamic load reaches the maximum value in a very short time; the settlement attenuation stage, due to the influence of the change of train dynamic load; and the settlement stable stage, in which the settlement attenuation decreased to a certain value [44].

**Figure 15:**
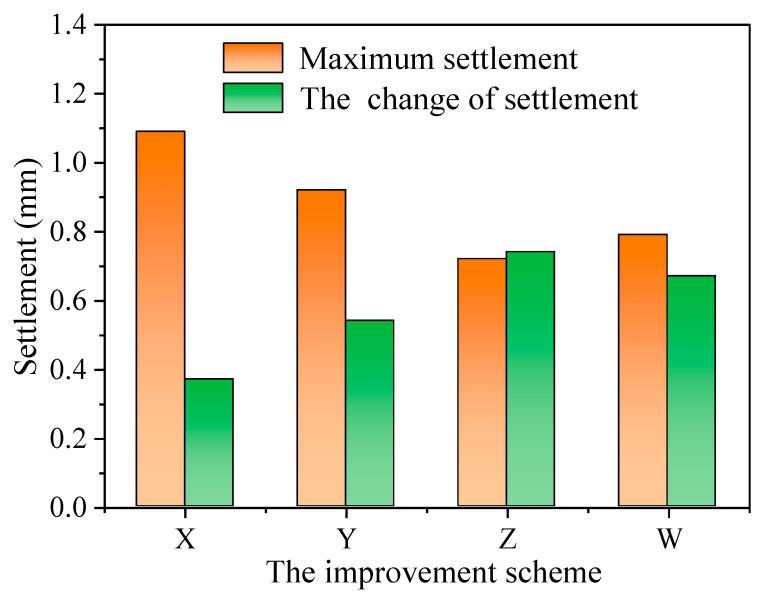
Changes in the maximum settlement of each improvement scheme.

The subgrade settlement and its variation under different improvement schemes were compared (Figure 15), and the results show that the compressive strength of improved loess has the most influence on the settlement of high-speed railway subgrade, followed by cohesion, and the influence of the internal friction angle is relatively little, indicating that the settlement of subgrade is more affected by the compressive capacity of soil compared with the shear strength of loess. Therefore, when considering the improvement of high railway foundation filler in loess area, the compressive strength of modified loess should be given priority under the premise of comprehensively considering the improvement of loess strength indexes.

Using the direct shear test, unconfined compressive test and numerical simulation analysis, the influence of single train passing on subgrade settlement was investigated. Considering that long-term settlement of loess subgrade was frequently found, which is attributed to the influence of long-term vibration load, the influence of long-term train vibration load on loess subgrade settlement would be studied in future research.

## 6. Conclusions

In this paper, a series of direct shear tests and unconfined compressive tests were conducted on the loess specimens improved by the composite material consisting of cement, polypropylene fiber and SCA-2 soil curing agent. The influence of the improvement scheme obtained from the test on the subgrade settlement was studied by numerical simulation. The following conclusions can be drawn:

(1) Cement, polypropylene fiber and SCA-2 soil curing agent can effectively improve the shear strength and compressive strength of loess, and the degree of influence of cement, polypropylene fiber and curing agent on the internal friction angle, cohesion and compressive strength is cement > fiber > curing agent.

(2) Based on the orthogonal test results and MIDAS numerical simulation method, the optimal improvement scheme for composite materials to improve loess was obtained: 16% cement content + 0.5% fiber content + 4% curing agent content.

(3) Through the numerical simulation calculation method, it is found that the compressive strength index of the improved loess has a significant impact on the subgrade settlement, and the optimal improvement scheme obtained can effectively decrease the settlement of high-speed railway subgrade under vibration load, and can effectively avoid safety accidents caused by cumulative subgrade settlement.

## Figures and Tables

**Table 1:** Basic physical indexes of loess.

*ρ_d_*	*W*	*ρ*	*G_S_*	*W_L_*	*W*_p_	Grain Size Fractions (%)		
						<0.005 mm	0.005–0.075 mm	0.075–0.05 mm
1.35	12	1.51	2.70	27.1	17.6	26.4	71.3	2.3

**Notes**: *ρ_d_* = dry density (g/cm^3^); *W* = natural water content (%); *ρ* = natural density (g/cm^3^); *G_S_* = specific gravity; *W_L_* = liquid limit (%); *W*_p_ = plastic limit (%).

**Table 2:** The main chemical composition of cement.

Raw Material	Chemical Composition (%)						
	CaO	Fe_2_O_3_	SiO_2_	Al_2_O_3_	MgO	SO_3_	LOI
Cement	63.89	4.53	22.78	5.46	0.79	1.31	1.24

**Table 3:** Physicomechanical characteristics of polypropylene fiber.

Type	Density (g/cm^3^)	Diameter (mm)	Tensile Strength (MPa)	Elastic Modulus (MPa)	Melting Point (°C)
Bundle monofilament	0.91	0.045~0.18	≥350	≥3600	165~175

**Table 4:** Test scheme.

Sample No.	Cement Content (%)	Fiber Content (%)	Curing Agent Content (%)	Sample No.	Cement Content (%)	Fiber Content (%)	Curing Agent Content (%)
1	0	0	0	14	8	0.75	0
2	0	0.25	4	15	8	1	4
3	0	0.5	8	16	12	0	12
4	0	0.75	12	17	12	0.25	16
5	0	1	16	18	12	0.5	0
6	4	0	4	19	12	0.75	4
7	4	0.25	8	20	12	1	8
8	4	0.5	12	21	16	0	16
9	4	0.75	16	22	16	0.25	0
10	4	1	0	23	16	0.5	4
11	8	0	8	24	16	0.75	8
12	8	0.25	12	25	16	1	12
13	8	0.5	16	—	—	—	—

**Table 5:** Results of variance analysis of internal friction angle.

	Class III Sum of Squares	Degrees of Freedom	Mean Square	F	*p*
Modified model	1492.3	12	124.4	5.0	0.005
Intercept	20,793.1	1	20,793.4	833.1	0.000
Cement	918.4	4	229.64	9.2	0.001
Fiber	490.2	4	122.6	4.9	0.014
Curing agent	83.6	4	20.9	0.8	0.527
Error	299.5	12	25.0		

**Table 6:** Results of variance analysis of the cohesion.

	Class III Sum of Squares	Degrees of Freedom	Mean Square	F	*p*
Modified model	23,171.4	12.0	1930.9	1.8	0.162
Intercept	106,268.4	1.0	106,268.4	98.7	0.000
Cement	13,730.7	4.0	3432.7	3.2	0.043
Fiber	3239.1	4.0	809.8	0.8	0.575
Curing agent	6201.6	4.0	1550.4	1.4	0.280
Error	12,918.0	12.0	1076.5		

**Table 7:** Results of variance analysis of the compressive strength.

	Class III Sum of Squares	Degrees of Freedom	Mean Square	F	*p*
Modified model	15,366.6	12.0	1280.6	10.0	0.000
Intercept	38,181.2	1.0	38,181.2	299.6	0.000
Cement	12,511.4	4.0	3127.9	24.5	0.001
Fiber	1606.6	4.0	401.7	3.2	0.048
Curing agent	1248.5	4.0	312.1	2.4	0.103
Error	1529.2	12.0	127.4		

**Table 8:** Calculation parameters of the finite element model.

Parameter	*h*	*c*	*φ*	*E*	*μ*	*γ*
Track board	0.3	—	—	325,00	0.18	26
Ballast bed	0.5	40	—	220	0.23	22
Surface layer of subgrade	1.5	30	20	200	0.32	22
Bottom layer of subgrade	3	42	28	150	0.32	21
Embankment (Loess)	3	26.21	13.7	21.07	0.4	17
(Scheme X)	—	117.35	35.78	64.87	0.37	18
(Scheme Y)	—	140.86	27.42	71.92	0.37	18
(Scheme Z)	—	135.66	30.43	85.37	0.37	18
(Scheme W)	—	131.17	32.36	79.46	0.37	18

**Notes**: *h* = thickness (m); *c* = cohesion (kPa); *φ* = internal friction angle (°); *E* = elastic modulus (kPa); *μ* = Poisson ratio; *γ* = bulk density (kN/m^3^).

## Data Availability

The data presented in this study are available upon request from the corresponding author.
